# Supplementary material for: Transcriptomic and Metabolomic Analysis Reveal the Effects of Light Quality on the Growth and Lipid Biosynthesis in Chlorella pyrenoidosa
Source: Biomolecules. 2024 Sep 10;14(9):1144. doi: 10.3390/biom14091144 (PMC11430191; doi:10.3390/biom14091144)
Supplement: Supplementary file 1 [file biomolecules-14-01144-s001.zip › biomolecules-3184173 - Supplementary Figures.pdf]

biomolecules

MDPI

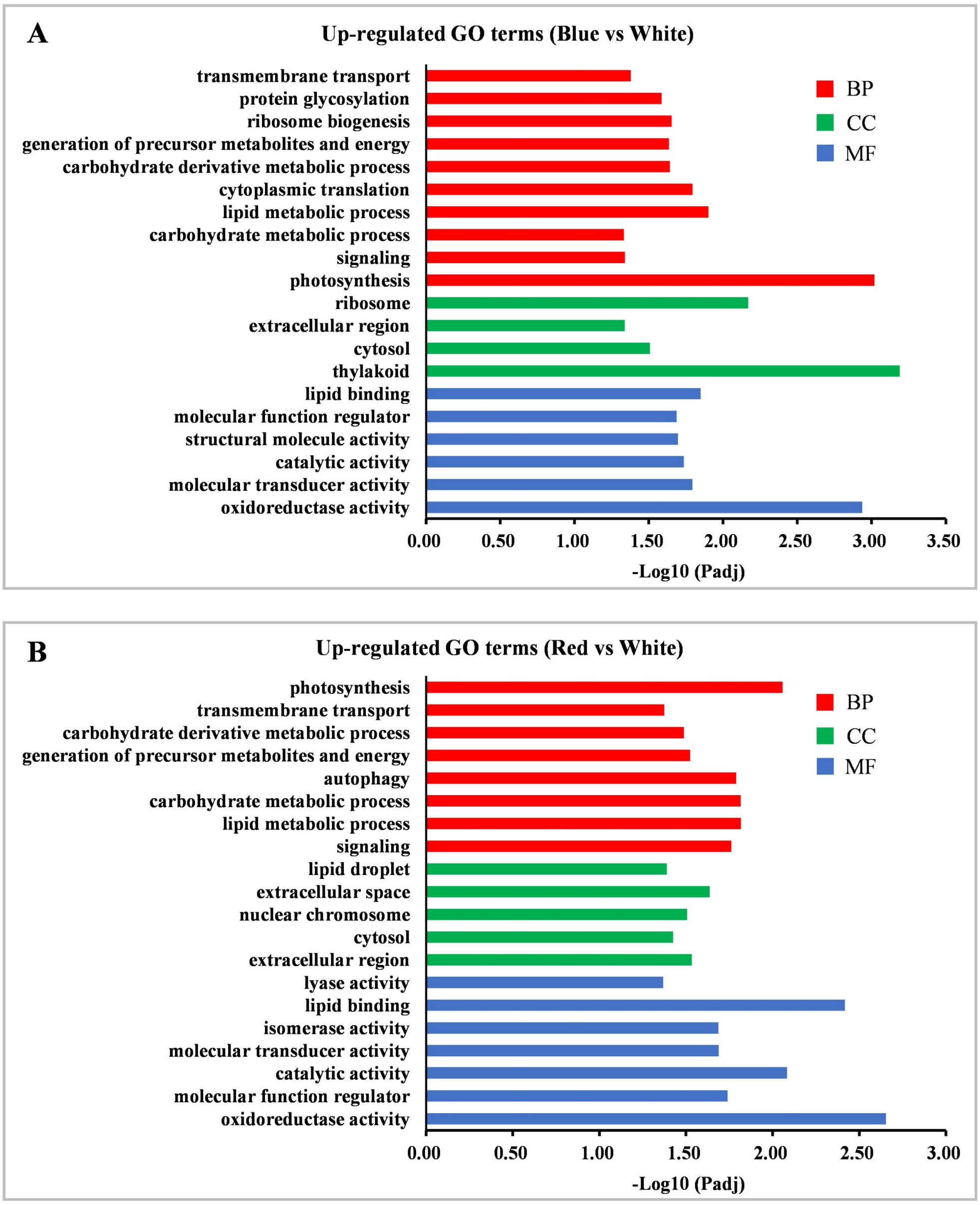

Figure S1. The 20 most significantly up-regulated GO terms. GO enrichment analysis of up-regulated gene in blue vs. white (A) and red vs. white (B). BP: biological process, CC: cellular component, and MF: molecular function.

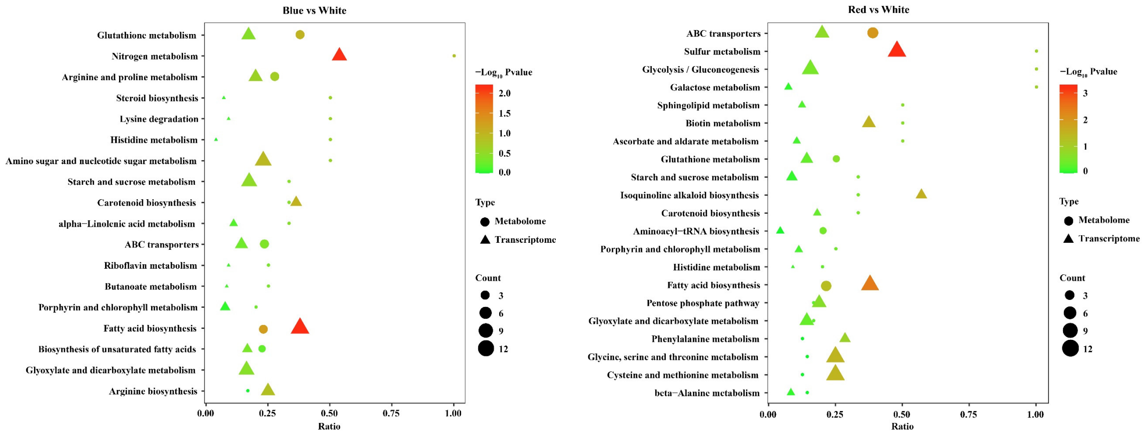

Figure S2. KEGG enrichment of differentially expressed genes and differential metabolites in blue vs. white and red vs. white group.

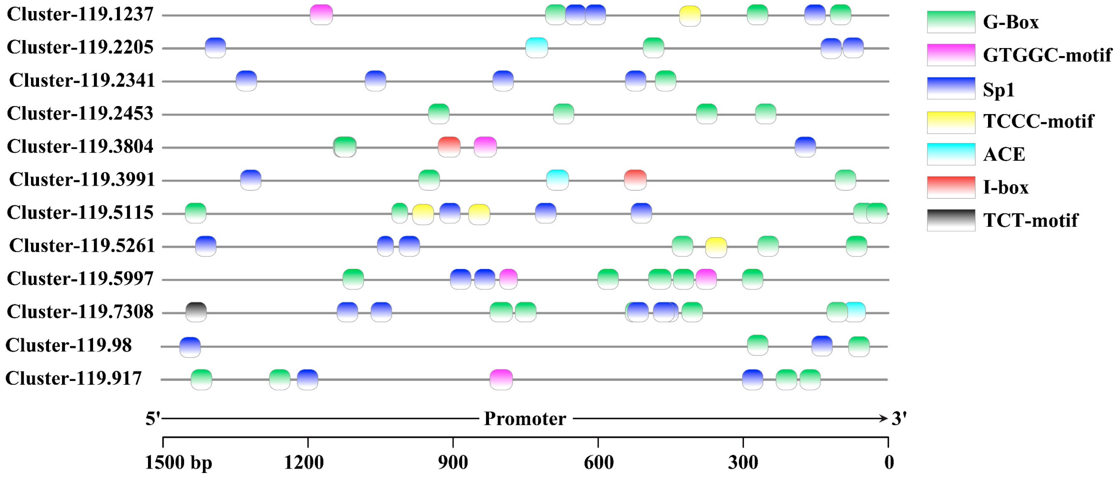

Figure S3. Promoter elements analysis of upregulated fatty acid genes. Promoter elements were analyzed in the PlantCARE online website, and light responsive elements were shown in the figure.

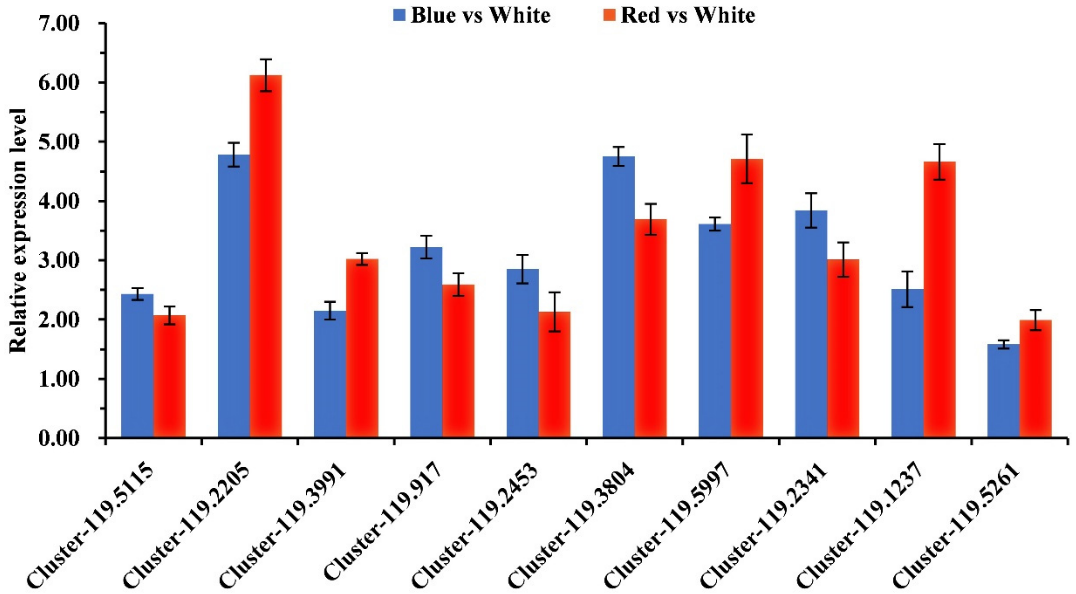

Figure S4. Gene expression levels of fatty acid biosynthesis genes in the real time PCR. The data were expressed as the mean ± SD of three independent experiments.
