# Supplementary material for: Transcriptomic and Metabolomic Analysis Reveal the Effects of Light Quality on the Growth and Lipid Biosynthesis in Chlorella pyrenoidosa
Source: Biomolecules. 2024 Sep 10;14(9):1144. doi: 10.3390/biom14091144 (PMC11430191; doi:10.3390/biom14091144)
Supplement: Supplementary file 1 [file biomolecules-14-01144-s001.zip › biomolecules-3184173 - tables.pdf]

biomolecules

Table S1. Primers used for qRT-PCR in this study.

Primer name	Sequence (5' to 3')	Length (bp)
CpActin F	GGCTACGTGGCGCTCGACTA	20
CpActin R	CGTCATGGAAGGCTGGAACAG	21
Cluster-119.5115 F	GATAGCCACCATCAAGGCCA	20
Cluster-119.5115 R	TCATTGGGCACCACGTTGAT	20
Cluster-119.2205 F	GCAGCAGCACGCCTTCC	17
Cluster-119.2205 R	GGTTCTTCTTGGCCTTGTCCT	21
Cluster-119.3991 F	CTGTGTTCTCTCGCCTGTCC	20
Cluster-119.3991 R	TCGGAGAAGTCGCCAAACAG	20
Cluster-119.917 F	TCGCTGGGCCTCAAAGTATC	20
Cluster-119.917 R	TCGCTGTCTGCCTTCTCTTC	20
Cluster-119.2453 F	GCAAAGCCCGCAGTTGTTC	19
Cluster-119.2453 R	AGGAAGTAGCCCTCCTCGTC	20
Cluster-119.3804 F	TTCTGCATCCCCTTTGCCAT	20
Cluster-119.3804 R	TGGAGATGGGGTAGTTGGGT	20
Cluster-119.5997 F	TTCGCCAACATCTACCCCAT	20
Cluster-119.5997 R	GCGTACCGCTTGTTCTCCTT	20
Cluster-119.2341 F	ATAGATGCTGTCGCGCTGG	19
Cluster-119.2341 R	CTCCCTCCCCCATCACAAAG	20
Cluster-119.1237 F	ACTTCTTCTTCGGTGGCGTG	20
Cluster-119.1237 R	CAAGGTCCTCGCCCACATAC	20
Cluster-119.5261 F	CACCTACTTCAACCTGGGGG	20
Cluster-119.5261 R	TTCGGTGTCGCAGCAGTAG	19

Table S2. Differentially expressed genes in the photosynthetic process.

Gene_ID	Blue vs. White		Red vs. White		KO Name	KO Description
	Log2 (Fold Change)	P-adj	Log2 (Fold Change)	P-adj		
**Photosynthesis**						
Cluster-119.170	6.34	1.73E-09	6.23	1.9E-10	PETF	Ferredoxin
Cluster-119.7052	2.07	2.46E-27	1.32	4.56E-13	PSBP	Photosystem II enhancer protein 2
Cluster-119.3653	1.96	4.69E-25	1.15	7.92E-10	PSAH	Photosystem I subunit VI
Cluster-119.5572	-2.66	5.28E-16	-4.89	2.18E-38	PSBS	Photosystem II 22kDa protein
Cluster-119.5851	1.78	2.90E-20	1.01	5.60E-09	PSAN	Photosystem I subunit PsaN
Cluster-119.5511	2.06	8.64E-43	1.65	2.06E-27	PETF	Ferredoxin
Cluster-119.6783	2.91	6.78E-36	1.91	6.51E-23	PETE	Plastocyanin
Cluster-119.5067	1.69	1.76E-40	1.12	2.68E-19	ATPF0B	F-type H+-transporting ATPase
Cluster-119.6123	1.06	6.42E-13	—	—	PSAF	Photosystem I subunit III
Cluster-119.6279	1.39	3.21E-21	—	—	PSAL	Photosystem I subunit XI
Cluster-119.4478	1.55	4.35E-16	—	—	PSAO	Photosystem I subunit PsaO
Cluster-119.5347	1.67	4.05E-34	—	—	PSB28	Photosystem II 13kDa protein
Cluster-119.4041	1.43	2.78E-13	—	—	PSAK	Photosystem I subunit X
Cluster-119.6456	1.75	1.84E-31	—	—	ATPF1G	ATP synthase, subunit gamma
Cluster-119.3739	1.03	3.46E-06	—	—	PSBP	Photosystem II enhancer protein 2
Cluster-119.6192	1.63	1.19E-14	—	—	PSBQ	Photosystem II enhancer protein 3
Cluster-119.5206	1.92	4.11E-32	—	—	PETJ	Cytochrome c6
**Photosynthesis-antenna proteins**						
Cluster-119.1465	3.34	1.34E-88	5.04	3.00E-99	LHCB1	Photosystem II chlorophyll a/b binding protein 1
Cluster-119.6037	2.34	1.42E-43	1.42	6.78E-24	LHCA1	Photosystem I chlorophyll a/b binding protein 1
Cluster-119.5444	3.07	4.67E-77	2.68	2.54E-117	LHCB1	Photosystem II chlorophyll a/b binding protein 1
Cluster-119.7358	2.30	7.86E-31	1.42	9.30E-20	LHCB2	Photosystem II chlorophyll a/b binding protein 2
Cluster-119.6257	1.80	5.79E-22	1.31	4.53E-16	LHCB4	Photosystem II chlorophyll a/b binding protein 4
Cluster-119.6063	2.64	1.32E-50	1.87	5.07E-26	LHCB2	Photosystem II chlorophyll a/b binding protein 2
Cluster-119.6459	2.26	5.33E-30	1.45	9.13E-18	LHCB5	Photosystem II chlorophyll a/b binding protein 5
Cluster-318.0	3.70	3.26E-23	2.93	1.05E-13	LHCB2	Photosystem II chlorophyll a/b binding protein 2
Cluster-119.6337	2.88	1.27E-57	2.06	3.69E-38	LHCB1	Photosystem II chlorophyll a/b binding protein 1
Cluster-119.6741	2.19	8.64E-17	1.07	2.89E-07	LHCB2	Photosystem II chlorophyll a/b binding protein 2
Cluster-119.7021	2.12	2.45E-26	1.23	1.44E-15	LHCB1	Photosystem II chlorophyll a/b binding protein 1
Cluster-119.7125	2.03	8.54E-49	1.17	6.82E-27	LHCA3	Photosystem I chlorophyll a/b binding protein 3
Cluster-119.7027	1.60	2.02E-26	—	—	LHCA4	Photosystem I chlorophyll a/b binding protein 4
Cluster-119.7728	1.18	2.26E-06	—	—	LHCA4	Photosystem I chlorophyll a/b binding protein 4
Cluster-119.6366	1.52	1.90E-18	—	—	LHCA4	Photosystem I chlorophyll a/b binding protein 4
**Carbon fixation in photosynthetic organisms**						
Cluster-119.7059	2.50	1.33E-29	1.16	1.50E-11	GAPA	Glyceraldehyde-3-phosphate dehydrogenase
Cluster-119.5862	1.61	5.72E-18	1.05	2.82E-09	PRK	Phosphoribulo kinase
Cluster-119.6446	1.78	2.20E-13	1.09	2.98E-06	E2.2.1.1	Transketolase
Cluster-119.1308	1.43	2.02E-04	1.69	1.23E-06	GOT2	Aspartate aminotransferase, mitochondrial

Cluster-119.8768	1.71	2.91E-20	1.18	1.06E-08	RPIA	Ribose 5-phosphate isomerase A
Cluster-119.4251	2.07	3.87E-41	1.15	7.74E-07	RBCS	Ribulose-bisphosphate carboxylase small
Cluster-119.5856	3.53	1.64E-66	2.54	8.98E-36	PGK	Phosphoglycerate kinase
Cluster-119.6654	1.96	2.62E-26	1.37	2.49E-14	ALDO	Fructose-bisphosphate aldolase, class I
Cluster-119.1193	2.40	2.11E-43	1.72	7.90E-24	TPI	Triosephosphate isomerase (TIM)
Cluster-119.5059	1.12	1.61E-07	—	—	RPE	Ribulose-phosphate 3-epimerase
Cluster-119.3867	1.10	7.27E-13	—	—	ALDO	Fructose-bisphosphate aldolase, class I
Cluster-119.7039	1.09	3.05E-09	—	—	RBCS	Ribulose-bisphosphate carboxylase small

Table S3. Differentially expressed genes in the up-regulated ribosome in blue vs. white.

Gene_ID	Log_2(Fold Change)	P-adj	KO Name	KO Description
Cluster-119.8542	1.02	3.76E-08	RP-L11	Large subunit ribosomal protein L11
Cluster-119.3676	2.09	1.74E-26	RP-L3	Large subunit ribosomal protein L3
Cluster-119.5081	1.42	1.62E-21	RP-L28	Large subunit ribosomal protein L28
Cluster-119.7801	1.20	7.44E-06	RP-L32	Ribosomal protein L32
Cluster-119.513	1.52	4.84E-03	RP-L4	Ribosomal protein L4
Cluster-119.411	3.20	8.29E-10	RP-S5	Ribosomal protein S5
Cluster-119.7928	2.56	8.66E-27	RP-S13	Ribosomal protein S13
Cluster-119.7852	1.14	1.00E-11	RP-L35	Large subunit ribosomal protein L35
Cluster-119.5504	1.08	1.97E-08	AOC3	Primary-amine oxidase
Cluster-119.6038	1.28	3.22E-08	PDR	ATP-binding cassette, subfamily G
Cluster-119.5961	1.20	1.21E-19	RP-L4	Large subunit ribosomal protein L4
Cluster-119.3072	1.64	2.42E-18	RP-L21	Large subunit ribosomal protein L211
Cluster-119.4360	1.96	3.54E-19	RP-S11	Ribosomal protein S11
Cluster-119.3594	1.37	2.13E-21	RP-L18	Large subunit ribosomal protein L18
Cluster-119.7265	2.22	1.11E-41	RP-S6	Small subunit ribosomal protein S6
Cluster-119.2911	1.06	2.18E-12	RP-L24	Large subunit ribosomal protein L24
Cluster-119.4964	1.78	3.61E-27	RP-S21	Small subunit ribosomal protein S21
Cluster-119.3484	1.58	1.43E-24	RP-L17	Large subunit ribosomal protein L17
Cluster-119.4961	1.03	2.58E-13	RP-L6	Large subunit ribosomal protein L6
Cluster-119.3486	1.69	2.02E-27	RP-L13	Large subunit ribosomal protein L13
Cluster-119.3049	1.31	1.66E-15	RP-S5	Small subunit ribosomal protein S5
Cluster-119.1121	2.65	8.90E-25	ACP7	Acid phosphatase type 7
Cluster-119.2175	2.00	2.20E-13	RP-L29	Ribosomal protein L29
Cluster-119.5159	1.03	6.28E-11	ASD	Semialdehyde dehydrogenase
Cluster-119.3921	1.61	2.98E-23	ADT	Arogenate/prephenate dehydratase
Cluster-119.6918	3.54	9.61E-96	RP-S14	Ribosomal protein S14
Cluster-119.4249	1.54	6.64E-09	EFTs	Elongation factor Ts
Cluster-119.8399	1.67	1.47E-13	RP-S13	Ribosomal protein S13
Cluster-119.6559	1.80	1.32E-16	RP-S5	Ribosomal protein S5
Cluster-119.5068	2.92	2.00E-115	CDO1	Cysteine dioxygenase
Cluster-119.7468	2.40	1.91E-57	RP-L19	Ribosomal protein L19
Cluster-119.5601	2.09	1.13E-28	RP-S6	Ribosomal protein S6
Cluster-119.3314	1.35	1.58E-21	RP-S20	Small subunit ribosomal protein S20
Cluster-119.5527	1.58	2.79E-14	EFG	Elongation factor G
Cluster-119.1214	1.83	2.38E-12	eIF-2B	Translation initiation factor EIF-2B
Cluster-119.697	1.06	1.98E-03	eIF-4E	Eukaryotic initiation factor 4E
Cluster-119.8957	1.22	2.19E-03	EF3	Elongation factor 3

Table S4. Differentially expressed genes in fatty acid biosynthesis process.

Gene ID	Blue vs. White		Red vs. White		KO Name	KO Description
	Log_2 (Fold Change)	P-adj	Log_2 (Fold Change)	P-adj		
Cluster-119.5115	1.48	5.62E-13	1.37	1.04E-13	FabF	3-oxoacyl-[acyl-carrier-protein] synthase I
Cluster-119.2205	2.68	3.43E-75	3.14	8.08E-100	FAB2	Acyl-[acyl-carrier-protein] desaturase
Cluster-119.3991	1.00	4.31E-08	1.31	1.99E-14	FAB2	Stearoyl-[acyl-carrier-protein] 9-desaturase 2
Cluster-119.917	1.89	2.97E-12	1.59	3.94E-08	FabG	3-oxoacyl-[acyl-carrier-protein] reductase
Cluster-119.2453	1.54	2.28E-32	1.76	9.92E-33	ACSF3	Probable CoA ligase CCL8
Cluster-119.3804	2.23	2.17E-43	2.04	4.91E-35	FabF	3-oxoacyl-[acyl-carrier-protein] synthase II
Cluster-119.5997	2.45	9.97E-71	2.36	5.41E-75	FabI	Enoyl-[acyl-carrier-protein] reductase [NADH]
Cluster-119.2341	1.49	1.66E-15	1.31	1.10E-12	FabF	3-oxoacyl-[acyl-carrier-protein] synthase

Cluster-119.1237	1.50	9.66E-15	1.64	4.00E-17	FabZ	3-hydroxyacyl-[acyl-carrier-protein] dehydratase
Cluster-119.5261	1.31	3.01E-15	1.84	7.32E-30	FATA	Fatty acyl-ACP thioesterase A
Cluster-119.98	2.10	4.44E-02	3.66	3.76E-02	FabF	3-oxoacyl-[acyl-carrier-protein] synthase I
Cluster-119.7308	1.12	8.31E-03	1.26	6.16E-04	ACAA1	Acetyl-CoA acyltransferase 1

Table S5. Differentially expressed metabolites in fatty acid biosynthesis process.

Metabolite ID	Blue vs. White		Red vs. White		Name	Formula
	Log_2 (Fold Change)	P-adj	Log_2 (Fold Change)	P-adj		
Com_9864_neg	0.59	0.0127	1.12	0.0363	Tetradecanoic acid	$C_{14}H_{28}O_2$
Com_2682_neg	1.94	0.0200	2.81	0.0096	Hexadecanedioic acid	$C_{16}H_{30}O_4$
Com_5906_pos	1.35	0.0415	1.07	0.0318	Octadecanoic acid	$C_{18}H_{36}O_2$
Com_154_pos	1.70	0.0418	1.83	0.0258	Docosahexaenoic acid	$C_{22}H_{32}O_2$
Com_423_pos	1.86	0.0432	1.34	0.0372	Linoleic acid	$C_{18}H_{32}O_2$
Com_1195_pos	3.54	0.0334	1.35	0.0261	Docosatrienoic acid	$C_{22}H_{38}O_2$
Com_12032_neg	1.08	0.0139	1.09	0.0147	FAHFA (16:1/18:3)	$C_{34}H_{58}O_4$
Com_354_pos	3.53	0.0271	0.96	0.0082	Adrenic acid	$C_{22}H_{36}O_2$
Com_3970_neg	1.05	0.0129	1.52	0.0369	Pentadecanoic acid	$C_{15}H_{30}O_2$
Com_602_pos	0.64	0.0147	1.54	0.0234	Eicosapentaenoic acid	$C_{20}H_{30}O_2$
